# The "Excellence in Translational Medicine" and "Bedside-to-Bench" Awards 2008-09

**DOI:** 10.1186/1479-5876-8-95

**Published:** 2010-10-13

**Authors:** Richard J Ablin, Francesco M Marincola, Pier Giorgio Natali

**Affiliations:** 1 Department of Pathology, University of Arizona College of Medicine; Arizona Cancer Center and BIO5 Institute, Tucson, AZ 85724, USA; 2 Infectious Disease and Immunogenetics Section (IDIS), Department of Transfusion Medicine, Clinical Center, and trans-NIH Center for Human Immunology (CHI), National Institutes of Health, Bethesda, MD 20892, USA; 3 CINBO Laboratories, "G.d'Annunzio" University, Chieti, Italy

## 

In a continuing endeavor to recognize outstanding contributions in the field of translational medicine, the Editorial Board of the Journal of Translational Medicine (JTM) established "The Excellence in Translational Medicine Award" in 2006 [1]. With the thought to also recognize excellent studies, defined as those exclusively based on the study of human subjects, the Editorial Board has further established "The Bedside-to-Bench Award" in 2008 [2].

The recipients of "The Excellence in Translational Medicine" and "Bedside-to-Bench" Awards will each receive a $5,000 prize sponsored by Medistem http://www.medisteminc.com/ and the Harry J. Lloyd Fund, respectively. The funds received from each Award are to be used to cover expenses for any meeting sponsored by a non-for-profit organization that is relevant to the goal of translational medicine and research.

Twenty-three papers nominated, including 13 highly accessed, from investigators representative of ten countries of five continents, covering a wide range of disciplines published in JTM between 1 July 2008-30 June 2009 were evaluated. For this purpose, an Award Committee* comprised of eight members of the Editorial Board selected and co-chaired by Richard J. Ablin (University of Arizona College of Medicine and the Arizona Cancer Center, Tucson, AZ) and Pier Giorgio Natali (CINBO Laboratories, "G.d'Annunzio" University, Chieti, Italy) was formed. The initial National Institutes of Health Scoring System of 1-5, with 1 = Outstanding and 5 = Poor, were used with the papers being evaluated with regard to their:

• Scientific merit

• Originality

• Clarity

• Relevance to the purposes of translational medicine and research (and in "The Bedside-to-Bench Award" to direct study of human subjects)

• Research design

• Methodology

## Excellence in Translational Medicine Award

In the paper by Hye-Won Chung [3], recipient of the "Excellence in Translational Medicine Award for 2008-09," Doctor Chung and colleagues of Yonsei University College of Medicine (Seoul, Korea) and the NIH (Bethesda, MD) have demonstrated a correlation between the serum levels of high mobility group protein box-1 (HMGB1) and the clinical and pathological characteristics of patients with gastric cancer (GC) and its suggested role therein as a biomarker.

Part of a group of chromosomal proteins known as the high mobility group (HMG) encoded by the HMBG1 gene, they are functionally involved in transcription, replication, recombination and DNA repair. HMGB1, a member of the HMG family of proteins, has been demonstrated to serve as a cytokine mediating lethal systemic inflammation via its extracellular release from activated monocytes/macrophages and cells undergoing necrosis.

mRNA levels of HMGB1 are known to be overexpressed in tissue in the majority of patients with GC and associated with tumour invasiveness and metastasis. However, evaluation in tissue requires invasive techniques, i.e., endoscopy and biopsy. Knowledge that HMGB1 is released as a cytokine into the extracellular microenvironment, suggested to Chung et al. that evaluation in serum might be useful.

Using an ELISA assay, Chung et al. [3] validated measurement of HMGB1 as a serological biomarker for GC and demonstrated for the first time that serum HMGB1 levels are significantly and sequentially increased in GC in accordance with disease progression.

## Bedside-to-Bench Award

Antiretroviral therapy (ART) in HIV-infected patients, particularly children, has resulted in increased survival. However, as discussed in the paper by Raffaele Badolato [4], recipient of the "Bedside-to-Bench Award 2008-09," and co-workers of the University of Brescia (Brescia, Italy), poor adherence to prescriptions and the high rates of virus replication, characteristic of perinatal HIV-infection have been noted to contribute to higher virological set points in children vs. adults and lower rates of attainment of undetectable viral loads. Therefore, the need for improved correlates of immune reconstitution and early predictors of AR failure in HIV-infected children.

Albeit, blood dendritic cells constitute less than 1% of total peripheral blood mononuclear cells, they exert relevant protection to pathogens by: i) producing IL-12 and interferon-alpha (IFN-α) and ii) inducing T-cell immunity via presentation of pathogen-specific antigens on their cellular surface. Additionally, IFN-α decreases HIV replication by induction of IFN-stimulated genes, including Myxovirus resistance 1, which encodes for the Myxovirus resistance protein A (MxA). MxA and quantification thereof as a biomarker, have been shown to capable of inhibiting several viruses, including HIV.

With the foregoing in perspective, the study by Badolato et al. [4] provides an exemplarly example of translational research. Therein, they utilized real-time PCR for measurement of MxA mRNA, a marker for the response to IFN therapy, to monitor the presumptive unresponsiveness of ART in perinatally HIV-infected patients; and demonstrated that analysis of MxA may be a valuable tool for the management of ART in perinatal HIV-infection.

With congratulations to Hye-Won Chung [3] and to Raffaele Badolato and their respective co-workers, the *3rd *"Excellence in Translational Medicine" and 2nd "Bedside-to-Bench" Awards are now history. We are hopeful these Awards will serve to encourage other investigators devoted to improving the "bench-to-bedside" and "bedside-to-bench" concepts of translational medicine and respective initiatives.

*"Excellence in Translational Medicine and Bedside-to-Bench Awards Committee": Richard J. Ablin (Co-Chairman); Howard L. Kaufman; Bruce Litman; Pier Giorgio Natali (Co-Chairman); Hideho Okada; Michael Perricone; Rja K. Puri; Noriyuki Sato.
